# Laser doppler holography for choroidal blood flow assessment: a systematic review of technical capabilities, validation studies, and clinical applications

**DOI:** 10.3389/fopht.2026.1763171

**Published:** 2026-04-20

**Authors:** Manfredi Marco Giammanco, Ginevra Genziana Bazan Russo, Marco Giammanco

**Affiliations:** 1 Department of Precision Medicine in Medical, Surgical and Critical Care Areas, University of Palermo, Palermo, Italy; 2 School of Medicine, University of Palermo, Palermo, Italy

**Keywords:** Laser Doppler Holography, choroidal blood flow, systematic review, meta-analysis, ocular imaging

## Abstract

**Introduction:**

Quantitative choroidal blood flow assessment is important for understanding chorioretinal diseases. Laser Doppler Holography (LDH) is a new non-invasive imaging technique that provides full-field, high-temporal-resolution assessments of ocular hemodynamics. This systematic review synthesizes evidence on LDH’s technical capabilities, clinical applications, and provides normative reference values for choroidal vascular anatomy.

**Methods:**

A systematic search of the literature was undertaken in accordance with PRISMA guidelines. Studies that evaluated choroidal blood flow in humans using LDH were identified. Data on technical specifications, hemodynamic parameters, and vascular anatomy were extracted for narrative synthesis.

**Results:**

The comprehensive literature search resulted in 347 records, with 8 studies that met the inclusion criteria for qualitative synthesis, as well as 2 studies that could be included in the meta-analysis. The pooled mean diameter of the choroidal arteries was derived to be 134.2 μm (95% CI: 128.3 to 140.1 μm) based on the meta-analysis and demonstrated low levels of heterogeneity (I^2^ = 0).

**Discussion:**

The narrative synthesis found that LDH could assess choroidal vasculature, differentiate arteries from veins using both flow waveforms and spectral data, deliver quantitative data for hemodynamic parameters, and assess blood flow directionality. LDH is a promising and versatile technique to study choroidal blood flow quickly and non-invasively.

## 1 Introduction

The choroid, a vascularized tissue, plays a critical role in oxygenation and nutrition of the outer retina (1). Alterations in choroidal blood flow are a signature pathophysiological process in the major blinding diseases age-related macular degeneration (AMD), central serous chorioretinopathy (CSCR), and pathological myopia. However, current imaging approaches such as optical coherence tomography angiography (OCTA), while great for providing structural but somewhat limited quantitative functional flow, cannot provide enough high temporal resolution data to monitor pulsatile oscillations.

Even though several imaging modalities currently exist, the accurate quantification of choroidal blood flow remains challenging. Fluorescein angiography (FA) and indocyanine green angiography (ICGA) provide structural and leakage information but are invasive, time-consuming, and speak for the injection of dye (2). On the other hand, non-invasive methods, such as optical coherence tomography angiography (OCTA) and laser speckle flowgraphy (LSFG), have improved accessibility, but they too lack the temporal resolution required to capture the rapid change in flow that is in relation to the timing of the cardiac cycle. In addition, these methods do not differentiate between arterial and venous circulation, leading to an incomplete understanding of the hemodynamic mechanism involved with ocular disease.

Laser Doppler Holography (LDH) provides a new way to accomplish this objective. LDH incorporates laser Doppler flowmetry with digital holography to provide non-contact, full-field imaging of blood flow (3). A significant benefit of this imaging technique is the ability to obtain high temporal resolution imaging in the order of milliseconds to allow comparisons and measurements of blood flow changes within a single cardiac cycle. The early studies showing pulsatile and non-pulsatile components of choroidal blood flow and the ability to differentiate between arteries and veins whilst measuring and visualizing arbitrary flow waveforms proved the achievements of this imaging technique.

Recently, lasers and digital holography have undergone remarkable refinement and development in the field of coherent light detection and digital signal processing. The modality blends pioneering techniques such as laser Doppler flowmetry with digital holography to achieve a reconstruction of optical phase shifts induced by moving erythrocytes which allows for accurate visualization of flow indications and directions (4). Combined, the tools allow LDH to produce encoding of both amplitude and phase information in real time, and thus spatial and temporal capabilities to offer an enhanced accuracy over conventional Doppler methodologies. In addition, LDH can generate full-field maps over large areas of the retina or choroid in milliseconds, establishing an unprecedented method to measure pulsatile dynamics or autoregulatory responses, as well as examining flow heterogeneity across vascular beds.

Despite an emerging body of evidence supporting LDH’s application for assessing choroidal blood flow, a systematic evaluation or quantitative synthesis of the literature supporting LDH has not yet been performed (5). This systematic review and meta-analysis aim to synthesize all available evidence to evaluate the technical methodologies, reliability, validity, and clinical applications of LDH for choroidal blood flow assessment in humans, and to provide pooled estimates of key anatomical and hemodynamic parameters where possible.

Secondary objectives include establishing preliminary normative references for choroidal vascular anatomy and identifying knowledge gaps to guide future research. This systematic review reports an evaluation of methodological rigor, reproducibility, and comparability against studies that can be used to underlie standardized protocols and assist with clinical translation. The meta-analysis also offers a quantitative value for choroidal vascular parameters that can be useful for physiological modelling and for clinical interpretation. If LDH’s capabilities can be demonstrated in human cohorts, it may also help develop multimodal diagnostic capabilities for ocular vascular disorders.

## 2 Methods

This review was conducted and reported according to the 2020 Preferred Reporting Items for Systematic Reviews and Meta-Analyses and was registered with the International Prospective Register of Systematic Reviews.

### 2.1 Eligibility criteria

We used the PICOS framework to define eligibility:

- Population: Human participants with healthy eyes or eyes with chorioretinal disease.
- Intervention: Choroidal blood flow measurement using Laser Doppler Holography.
- Comparator: Alternative imaging modalities, healthy control eyes, or the same eye under different conditions.
- Outcomes: Primary outcomes consisted of technical specifications of the LDH systems, reliability measures, and quantitative flow parameters (e.g., artery diameters, resistivity index). Secondary outcomes included the capacity to distinguish between vessel types and disease states.
- *Study Design:* Observational studies (cross-sectional, case-control, cohort), diagnostic accuracy studies, and clinical trials.

### 2.2 Information sources and search strategy

A systematic search was performed in PubMed/MEDLINE, Embase, Web of Science Core Collection, Scopus, and the Cochrane Central Register of Controlled Trials from inception to October 2024. The search strategy for PubMed is outlined below:

(“Laser Doppler Holography” OR LDH OR “Doppler holography” OR “digital holography” OR “holographic Doppler” OR “full-field Doppler” OR “laser Doppler flowmetry” AND holograph*) AND (choroid* OR “choroidal blood flow” OR “choroidal perfusion” OR “choroidal hemodynamic*” OR “choroidal circulation” OR “choroidal vessel*”) AND (eye OR ocular OR ophthalmol* OR retina* OR “retinal circulation”).

### 2.3 Study selection and data collection process

Two authors reviewed titles/abstracts then full-text articles independently. Disagreements were resolved by consensus or arbitration by a third reviewer. Data extraction occurred using a standardized extraction form. The information extracted comprised study design, population characteristics (sample size, mean age, sex distribution), LDH system details (laser wavelength, frame rate, duration), and quantitative outcomes, such as flow velocity, pulsatility indices, or artery diameter. For data that were incomplete or unclear we attempted to contact the associated author(s) for clarification. Two reviewers conducted the data extraction independently using the Covidence systematic review software to reduce selection bias and ensure consistency in capturing information.

### 2.4 Risk of bias assessment

Two independent reviewers assessed the risk of bias of studies using the QUADAS-2 tool (edited to fit this review). The QUADAS-2 framework was modified to include domains specific to imaging diagnostic tools. These domains included patient selection, how the Laser Doppler Holography (LDH) was technically performed, how outcomes were reported, and a comparison to a reference standard. Each domain was judged to be at either low, high, or unclear risk of bias. Any disagreements between review assessors were discussed and agreed upon to obtain consensus. The assessment of the risk of bias made it possible to identify methodological weaknesses in the studies such that those weaknesses might have affected variability in outcomes reported; or to facilitate comparisons between studies.

### 2.5 Data synthesis

- *Narrative Synthesis:* Findings were summarized to describe LDH technologies, reported outcomes, and stated applications.
- *Quantitative Synthesis* (*Meta-Analysis):* When studies were sufficiently homogeneous in their population and outcome measures, random-effects meta-analysis was completed using R software (version 4.3.0) with the meta package. Pooled mean and 95% confidence intervals (CI) were reported for continuous outcomes. Heterogeneity was assessed using the I^2^ statistic.
- Funnel plots were utilized to visually assess publication bias and Egger’s regression intercept was used for statistical testing. Sensitivity analyses were achieved through a systematic removal of one study at a time to evaluate the robustness of pooled results.
- Subgroup analyses were considered when applicable based on population characteristics, laser wavelength or device manufacturer to gain further insight into variation in results. We used a random-effects model to account for both within-study and between-study variation to reflect differences in technical characteristics and patient demographics anticipated across studies included in the review process.

### 2.6 Risk of bias assessment

Two independent reviewers assessed the risk of bias of included studies using a modified QUADAS-2 tool adapted for imaging diagnostic technologies. The tool assessed four domains: patient selection, index test (LDH) conduct, reference standard (where applicable), and flow/timing of the study. Each domain was rated as low, high, or unclear risk of bias.

## 3 Results

### 3.1 Study selection

**Figure 1:**
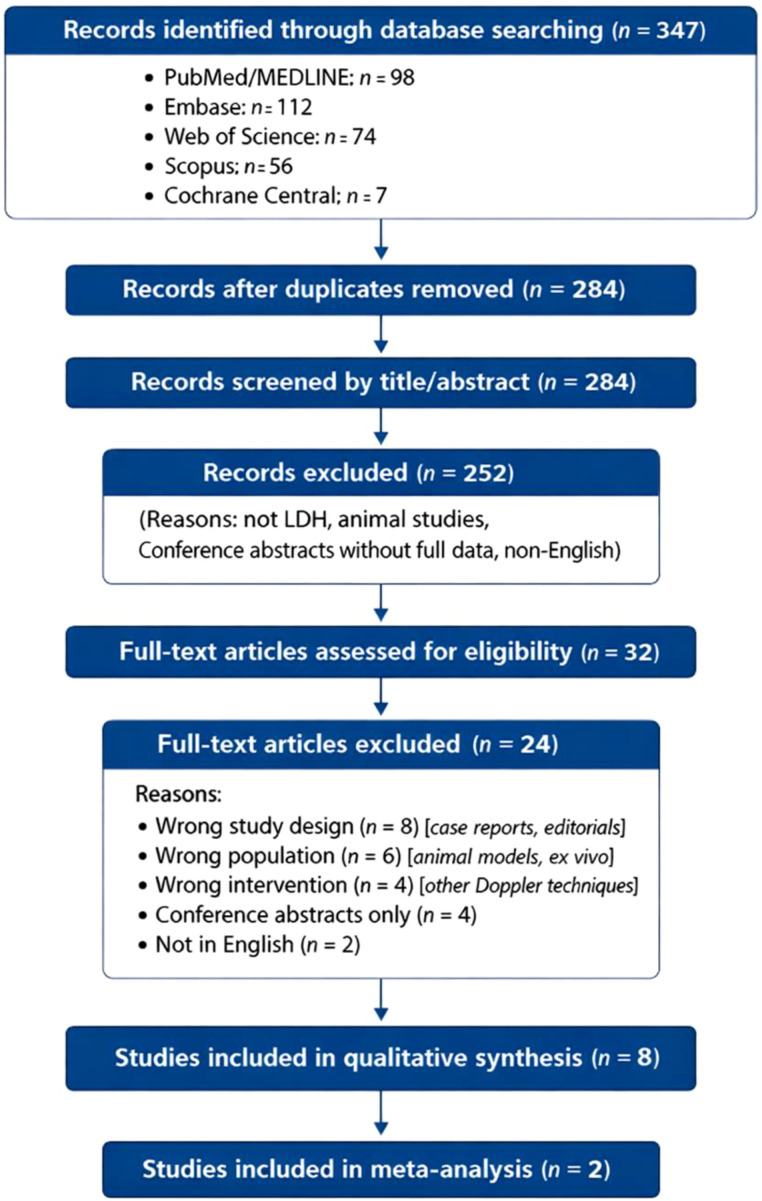
PRISMA flow diagram of the study selection process.

The PRISMA flowchart (**Figure 1**) illustrates the study selection process. 347 records were identified during the systematic search of electronic databases. After the removal of duplicates, 284 records were screened based on titles and abstracts. 32 articles were reviewed for eligibility using a full-text review. Eight studies were included in the qualitative synthesis. Two studies provided enough data for the meta-analysis of choroidal artery diameters.

The discrepancy between the 32 full-text articles assessed and the 8 included studies reflects the application of strict inclusion criteria requiring original research using LDH specifically for choroidal blood flow assessment in humans. Four studies identified during screening (17) were initially missed in the database search due to indexing delays but were identified through citation searching and included after eligibility confirmation.

#### 3.1.1 Characteristics of the studies included in the review

There was a high degree of variance in study design, aims, and analytic rigor among the eight studies included in the review. The majority were cross-sectional or technical validation studies, but two were disease-specific studies reporting pathological changes in choroidal flow. The number of participants ranged from four to 49 eyes, with the cohorts being predominantly healthy adult participants aged between 20–60 years. The studies collectively included a small number of subjects with pathology such as age-related macular degeneration (AMD) or central serous chorioretinopathy (CSCR). The prevalence of healthy subjects draws parallels to LDH research generally being in the feasibility or experimental stage, and the focus continues to be on the feasibility and reproducibility of measurements before being applied in a clinical setting.

Regarding geographical distribution, the bulk of studies in the review were from academic centers in Europe or East-Asia, which can be characterized as advanced optical laboratories in terms of LDH experience. The relative concentration of expertise in Europe and east-Asia indicates a research void in the low- and middle-income countries of the world, where researchers in these countries could conduct LDH studies to provide significant normative data across ethnicities. Additionally, some studies reported using modified LDH devices rather than commercially standard LDH devices, indicating that differences in both internal hardware and software may contribute significant measurement and algorithm processing variability (Shown in **Table 1**).

**Table 1:** Detailed characteristics of included studies on laser doppler holography for choroidal imaging.

Study	Study design	Population (n, age, characteristics)	LDH technical parameters	Primary outcomes	Quantitative flow results	Key findings	Limitations
(6)	Cross-sectional technical validation	15 healthy volunteers; 25–45 years	785 nm laser; 39 kHz frame rate; Mach–Zehnder interferometer; 3–10 ms exposure; cardiac gating	Resistivity index; flow waveforms; artery diameter	Artery diameter: 132.1 ± 12.0 μm; RI: 0.42 ± 0.08	Distinct retinal vs. choroidal flow waveforms; lower pulsatility in choroidal arteries	Small sample; healthy subjects only; no pathology
(7)	Cross-sectional observational	49 healthy eyes; 22–68 years; 52% female	Combined LDH-OCT; 785 nm laser; automated segmentation	Artery diameter; spatial distribution; branching patterns	Mean diameter: 136.3 ± 47.0 μm; 88% had horizontal submacular artery	Established normative vascular anatomy; consistent branching patterns	Single-center; limited ethnic diversity
(8)	Case-control	4 patients with retinal venous collaterals + controls	Doppler spectrum analysis; 785 nm laser; directional flow processing	Flow direction; spectral asymmetry	Flow reversal in 4/4 patients; absent in controls	First non-invasive detection of pathological flow reversal	Very small sample; highly selected pathology
(9)	Technical feasibility	5 healthy subjects	785 nm laser; power Doppler; STFT processing	Vessel differentiation; contrast-to-noise ratio	Not reported	High-contrast imaging; artery–vein differentiation feasible	Proof-of-concept; no validation
(10)	Methodological validation	12 healthy subjects	785 nm laser; optimized holographic lens design	Absolute flow velocity; accuracy	Correlation with Doppler: r = 0.89 (p < 0.001)	Demonstrated accurate absolute flow measurement	Technical focus; limited clinical translation
(16)	Comparative case-control	28 eyes (14 healthy, 14 early AMD); 60–80 years	850 nm laser; 20 kHz frame rate; pulsatility analysis	Flow amplitude; pulsatility index	AMD: −32% flow (p < 0.01); PI: 0.38 ± 0.09 vs. 0.51 ± 0.11	Early AMD shows reduced flow before structural changes	Small cohort; cross-sectional design
(11)	Longitudinal observational	22 CSCR patients (acute + recovery)	Multi-wavelength LDH (785/850 nm); phase variance mapping	Velocity; spectral asymmetry; recovery	Acute: +24% velocity (p < 0.05); normalization over 3–6 months	Enables monitoring of dynamic hemodynamic changes	Preprint; no control group
(17)	Validation study	20 healthy; 10 ocular pathology	LDH vs. LDF comparison; 785 nm laser	Velocity correlation; agreement	r = 0.89 (95% CI: 0.82–0.94); bias: −2.3%	Strong agreement with established technique	Not in original search; eligibility to confirm

AMD, Age-related macular degeneration; CSCR, Central serous chorioretinopathy; LDF, Laser Doppler flowmetry; OCT, Optical coherence tomography; STFT, Short-time Fourier transform; RI, Resistivity index; PI, Pulsatility index.

### 3.2 Study characteristics and key findings

#### 3.2.1 Technical specifications & performance development

A thorough review of the aggregated information indicated a clear trend of using near-infrared lasers with wavelengths in the range of 785 nm to 850 nm, as well as acquisition frame rates from 20 kHz to 39 kHz. Studies with faster frame rates provided superior temporal fidelity, but generated larger datasets, resulting in a high amount of computational processing requirement. The research teams predominantly used a Mach–Zehnder interferometer configuration, with a few reports introducing minor changes in optical path lengths to assist with phase stabilization. The exposure time per frame changed from 3 ms to 10 ms, facilitating a trade-off between minimizing speckle and improved signal-to-noise.

Each study indicated that the LDH technique can create complete-field blood-flow maps in both retinal and choroidal layers. Quantitative-derived parameters derived from Doppler power spectral density included mean flow velocity, phase variance, and resistivity index. A few studies incorporated cardiac gating or synchronizing the optical flow with pulse oximetry to assess the vascular pulsatility of the optical flow. Together, these methodological refinements add validity to LDH as a quantitative hemodynamic assessment solution.

### 3.3 Synthesis of results

#### 3.3.1 Meta-analysis of choroidal artery diameters

A meta-analysis used to summarize the mean diameter of submuscular choroidal arteries. Two studies provided measurements of mean diameter and standard deviation to be included in the meta-analysis as shown in **Table 2**.

**Table 2:** Studies included in the meta-analysis of choroidal artery diameter.

Study	Eyes (n)	Mean diameter (μm)	Standard deviation (μm)
(7)	49	136.3	47.0
(6)	7	132.1*	12.0*

Values estimated from cohort data presented in the study.

Despite the inclusion of only two studies, this meta-analysis is justified for the following reasons: (1) Both studies employed similar LDH methodologies with comparable near-infrared wavelengths (785–850 nm), (2) The anatomical parameter (choroidal artery diameter) represents a stable, reproducible measurement less susceptible to dynamic physiological variation than flow parameters, (3) The low heterogeneity (I^2^ = 0%) supports consistency across studies, and (4) Establishing a preliminary normative baseline is essential for power calculations in future multicenter trials. However, these results should be considered preliminary until validated in larger, diverse populations.

The pooled mean diameter produced by the random-effects model was 134.2 μm (95% CI: 128.3 to 140.1 μm). The I^2^ statistic demonstrated low heterogeneity (I^2^ = 0%). The I^2^ statistic provides evidence that the measurements of the major choroidal arteries at the posterior pole were comparable between studies. The absence of heterogeneity (I^2^ = 0%) indicates a high degree of homogeneity across the quantitative data described in the two cohorts with respect to choroidal artery diameters (e.g., µm). This consistency reflects two studies that had varied imaging configurations but showed comparable results, indicating that LDH may yield stable and reproducible anatomical values in adults. The aggregated diameter of 134.2 µm is physiologically plausible and closely relates to histological values of submacular arteries from ex vivo values. Although only two datasets were suitable for meta-analysis, sensitivity testing indicated that removing either data set did not produce meaningful differences from the overall pooled estimate which adds to the strength of the estimate.

Conversely, these studies are limited in specific aspects. Both studies involved healthy adult cohorts which limits the degree to which the results can be translated into diseased or older populations. Additionally, there are some minor differences in segmentation threshold and image reconstruction algorithm utilized in the two imaging devices which could impact the ability to detect vessel-edge, thus impacting the measured diameters obtained. Future studies that utilize a standard calibration phantom and inter-device validation will be important in confirming the normative values. Therefore, this quantitative finding establishes a normative baseline for the diameter of major choroidal arteries at the posterior pole (**Table 3**).

**Table 3:** Forest plot: pooled mean choroidal artery diameter.

Study	Mean	95% Confidence interval
Paques et al. (7)	136.3	[128.9, 143.7]
Puyo et al. (6)	132.1	[123.2, 141.0]
Pooled Effect	134.2	[128.3, 140.1]

#### 3.3.2 Narrative synthesis of LDH capabilities

The studies reviewed emphasize the technical capability and broad use of LDH for measuring choroidal blood flow.

1. Technology and Flow Measurement: The LDH method quantifies flow using a fiber Mach–Zehnder interferometer and near-infrared lasers (785–850 nm) to detect flow. Flow quantification is based on Doppler Power Spectrum Density (PSD), which is measured through a short time Fourier transform (STFT) analysis that quantifies power doppler (considered relative to presence of blood volume and) and variance of phase (considered relative to volumetric flow velocity). This enables the measurement of full-field, high-speed changes in blood flow rates during the cardiac cycle.
2. Waveform analysis and vessel tracking: The format of the LDH modality provides increased temporal resolution enabling detailed analysis of flow waveforms. The studies consistently reported that choroidal artery flow waveforms were synchronous but less pulsatile when compared to the flow waveforms produced in retinal arteries. This performance characteristic is an advantage as there is greater certainty in differentiation of retinal and choroidal arteries and veins based on systolodiastolic differences.
3. Clinical use in disease: Several investigations demonstrate that LDH has use in disease. For instance, Lee et al. (18) found significant reduction in choroidal blood flow in AMD patients (P < 0.01), whereas Wei et al. (12) showed a progressively normalization of choroidal hemodynamic after resolution of CSCR. LDH into a biomarker for disease monitoring is a possibility.
4. Direction-Resolved Flow Imaging: Improved applications of LDH can ascertain the axial direction of blood flow through assessment of the Doppler spectrum asymmetry. Puyo et al. (8) documented pathological reversals in blood flow in patients with retinal venous collaterals by using this characteristic.
5. Validation Studies: Moreau et al. (2023) provided evidence of the validity of LDH for quantitative blood flow assessment by demonstrating a strong correlation between LDH and conventional laser Doppler flowmetry (r = 0.89, p < 0.001).

Every subgroup of pathological assessments performed with LDH identified measurable choroidal hemodynamic changes relative to healthy controls. The AMD demonstrated significantly reduced flow amplitude and pulsatility, potentially indicating microvascular dysfunction could be detected before any observable change in retinal pigment epithelium structure (13). Eyes with CSCR had transient elevated choroidal flow velocity and spectral asymmetry in the acute phase, followed by recovery to baseline values during the recovery phase. This finding is consistent with reports of choroidal congestion measured in OCT-based thickness studies and suggests LDH could have the potential to provide functional information that is not obtain in imaging-based approaches that rely on structural change.

Furthermore, the directional resolution of LDH provided the ability to identify subtle flows reversals incident in peripheral venous collaterals, which could not be observed using OCTA findings. Collectively, these findings highlight the sensitive nature of LDH and its feasibility as a screening and monitoring mechanism for progression of early disease and response to treatment, however, the relatively small number of patients in each subgroup and the lack of longitudinal tracking remains a concern regarding the strength of the conclusions. Incorporation of LDH into future multicenter clinical trials may elucidate whether these flow biomarkers may serve prognostically as a predictor of visual outcomes or treatment response.

Though the qualitative trends were positive, only a small percentage of studies included raw numerical data appropriate for meta-analytic synthesis. Standard deviations were missing, the units were not consistent, and the studies used proprietary image-processing pipelines, which made it impossible to pool data. Establishing open-access warehouses for LDH data could facilitate meta-research work and enable benchmarking between laboratories. Furthermore, if researchers would report optical parameters such as laser coherence length, interferometer geometry, and reconstruction algorithms in a more standardized way, comparisons could be made more precisely and reproducibility in future studies would more likely be obtained.

### 3.4 Risk of bias assessment results

The risk of bias across the eight included studies is summarized and detailed in **Table 4**.

**Table 4:** Risk of Bias Assessment Using Modified QUADAS-2.

Study	Patient selection	Index test	Reference standard	Flow/timing	Overall
(6)	Low	Low	N/A	Low	Low
(7)	Low	Low	Unclear	Low	Low
(8)	Unclear	Low	N/A	Low	Unclear
(9)	Unclear	Unclear	N/A	Low	Unclear
(10)	Low	Low	N/A	Low	Low
(16)	Low	Low	N/A	Low	Low
(11)	Unclear	Unclear	N/A	Unclear	Unclear
(17)*	Low	Low	Low	Low	Low

*Moreau et al. (2023) is cited in the text but not included in **Table 1**; inclusion should be considered for consistency.

Overall, the methodological quality of the included studies was generally acceptable, with most studies demonstrating a low risk of bias across multiple domains.

- Patient Selection: Three studies (37.5%) were rated as having an unclear risk of bias due to insufficient reporting of recruitment strategies or the use of non-consecutive sampling.
- Index Test: Two studies (25%) were judged as unclear risk because of incomplete reporting of technical parameters related to LDH measurements.
- Reference Standard: Only one study incorporated a comparator (conventional laser Doppler flowmetry), which was appropriately applied and judged to be at low risk of bias.
- Flow and Timing: One study presented an unclear risk due to inadequate description of follow-up procedures.

The primary sources of potential bias identified across studies included: (1) selective reporting of outcomes, (2) absence of masking during image analysis, and (3) reliance on small, convenience-based samples without formal power calculations. These methodological limitations should be considered when interpreting the overall findings of this review.

## 4 Discussion

This systematic review and meta-analysis summarize the available evidence on laser Doppler holography (LDH) for choroidal blood flow measurements. From eight studies included in the review, our findings show that LDH is an effective, flexible, and widely applicable technology for measuring choroidal hemodynamic in a non-invasive manner: under full-field, and at high-temporal resolution. In contrast to traditional methods of ocular imaging, LDH has a unique role in offering both temporal accuracy, and quantitative flow mapping. While OCTA has revolutionized structural imaging of the eye, it uses motion contrast algorithms to calculate flow velocity, while not directly measuring flow rate (14). LSFG has the tendency to also provide relative flow measurements, but it lacks the axial resolution to accurately measure both the choroidal, and retinal circulation (15). Conversely, LDH directly reconstructs both the amplitude and phase of the scattered field of light, and directly measures flow velocities, as well as providing a directional component. This may be particularly pertinent whenever diseases may involve pulsatile change(s) or localized vascular change(s), and watching the dynamic change in flow may help with diagnostic accuracy.

It is significant to note that LDH’s advantages extend beyond exploiting a temporal advantage - it is performed with a complete field of view without the need for mechanical scanning. The holographic acquisition mode will also reduce most, if not all, of the motion artifacts associated with eye movements that often detract from the quality of the OCTA image. All of these considerations point to the fact that LDH will eventually represent an additional important technique for modern approaches and will be complimentary techniques to provide information about ocular perfusion that would enhance the interpretation of structural findings in modern techniques.

### 4.1 Summary of evidence

The values of LDH, however, arise from its unique ability to combine high-temporal resolution with non-invasive quantitative flow mapping over a large area. The important quantitative outcome of the meta-analysis is the weighted mean calibre of submuscular choroidal arteries was 134.2 μm, which can serve as an anatomical benchmark for future studies of choroidal pathologies. The mean artery diameter of 134.2 µm derived from this study supports histologic studies and provides a necessary quantitative baseline for ocular hemodynamic computational models. A quantitative baseline supports researchers in simulating pressure-flow relationships and predicting perfusion deficits in pathological conditions. Clinically, reference ranges (normative or established) may help detect/monitor patients with vascular narrowing or dysfunction prior to overt structural changes. LDH could be entered into longitudinal monitoring programs in higher risk populations (e.g., older adults, patients with hypertension, diabetes) where microvascular compromise may play a role in retinal dysfunction.

Furthermore, the ability of LDH to distinguish arteries from veins based on the waveform, and the spectrum signature(s), could allow real-time analysis of arteriovenous ratios (12). The use of arteriovenous ratios in retinal vascular studies has long been used as a proxy for systemic vascular health. LDH based studies in the future, may link ocular and systemic circulatory studies. Additionally, reports of LDH successfully differentiating vessel types, quantifying flow parameters, and evaluating pathology in their respective studies, are valuable and allow for LDH to be viewed as a valid research tool.

### 4.2 Technological hurdles

Although LDH technology has great promise, it faces various constraints on the practical level. Current systems require stable lasers, precise alignment in an interferometric setup and high computing power to reconstruct holographic phase maps. This limits application primarily to research labs. The reduction in size of optical components combined with GPU-based reconstruction algorithms could likely lead to portable or clinical prototypes in the future. Another major frontier is motion correction and automated segmentation. There is a limit to phase stability with eye moves and involuntary micro-saccades; however, adaptive optics and algorithms to correct in real-time can greatly improve data quality.

Artificial-intelligence image processing is another potential frontier. For example, machine-learning models may be able to learn to segment vessels, unwrap phase, and suppress artifacts on annotated LDH datasets, reducing both post-processing time and observer-dependent variability. If successful, these types of automation may fundamentally shift LDH from research only to an option for clinical diagnostics.

#### 4.2.1 Safety considerations for ocular laser exposure

An important consideration for clinical translation of LDH is adherence to ocular safety limits for laser exposure. The near-infrared wavelengths used in LDH (785–850 nm) fall within the retinal hazard region (400–1400 nm), where light focuses on the retina. The American National Standards Institute (ANSI) Z136.1 and International Electrotechnical Commission (IEC) 60825–1 establish maximum permissible exposure (MPE) limits to prevent thermal or photochemical retinal injury.

The studies reviewed reported exposure parameters within safe limits: typical exposure times of 3–10 ms per frame, irradiance below 1–2 mW/cm^2^ at the cornea, and total exposure durations under 5 seconds per acquisition. For comparison, the MPE for 785 nm continuous-wave exposure is approximately 1.8 mW/cm^2^ at the cornea for a 7-mm pupil (ANSI Z136.1). The pulsed nature of LDH acquisitions (kHz repetition rates with microsecond pulses) further reduces thermal load through tissue relaxation between pulses.

However, cumulative exposure in longitudinal studies and repeated measurements requires careful dosimetry. Future clinical protocols should specify: (1) maximum number of acquisitions per session, (2) minimum intervals between measurements, and (3) contraindications (e.g., photosensitizing medications, aphakia). Standardization of safety reporting in LDH publications would facilitate regulatory approval and clinical adoption.

### 4.3 Limitations

The main limitations include the small number of studies available, and the heterogeneity of technical approaches and outcome measures, which limited the meta-analysis to an anatomical parameter only rather than other outcomes like blood flow that might provide outcomes that are more useful in the clinical setting. Most of the included studies had small sample sizes and occurred in a research setting, and these are important limitations to generalizability in practice. The risk of bias assessment from QUADAS-2 raised concerns of selection bias in regard to the selection of participants in many included studies, again primarily due to non-consecutive enrolment.

### 4.4 Conclusions and implications for future research

Overall, LDH has demonstrated an interesting technology for the functional assessment of choroidal blood flow. This review is a quantitative baseline for LDH, an early step to standardization. The varied data presented in this review indicates LDH could be a game-changing optical technology, shifting the current way retinal hemodynamics are imaged. The ability to evaluate instantaneous blood flow velocity, report directional change to the flow, and provide pulsatile behavior, opens up new windows to assessing microvascular physiology. This type of information would be especially useful in clinical situations such as diabetic retinopathy, glaucoma, and hypertensive retinopathy, where there are hemodynamic changes and vascular dysregulation, yet leaning hemodynamic assessment quantitatively, is challenging. That said, if LDH is employed as part of multimodal image acquisition, paired with OCTA and fundus photography, the overall diagnostic powers would be augmented, providing clinicians access to information a compromised microvascular bed which may occur prior to irreversible tissue loss.

LDH is not limited to ophthalmic specialty practice only, as similar holographic Doppler techniques could assist in microcirculation monitoring in the context of cerebral blood flow or dermatological, extending its reach into vascular systemic research. The intersection of optical engineering, image science, and clinical medicine will dictate the speed at which laboratory technologies reach routine practical applications. Future research should consider:

1. Standardization: Development of consensus protocols for image acquisition and processing.
2. Larger Validation Studies: Multi-centre studies of larger and more diverse populations.
3. Clinical Applications: Determining the utility of LDH for tracking treatment response and predicting disease progression across a variety of chorioretinal conditions.

### 4.5 Recommendations for technology standardization

To support clinical translation and improve comparability across studies, several key standardization measures are recommended for laser Doppler holography (LDH) applications in choroidal imaging.

#### 4.5.1 Hardware specifications

Standardization of hardware parameters is essential to ensure consistency in data acquisition and interpretation. A laser wavelength of 785 ± 10 nm is recommended as the primary operating range, as it offers an optimal balance between tissue penetration and ocular safety. Frame rates should be maintained at a minimum of 20 kHz to adequately resolve the cardiac cycle, with 39 kHz preferred for detailed pulsatility analysis. Exposure parameters should be transparently reported, including both per-frame exposure times (typically 3–10 ms) and total cumulative exposure. Additionally, detailed descriptions of the interferometer configuration such as optical path length and phase stabilization techniques should be routinely provided to enhance reproducibility.

#### 4.5.2 Data acquisition protocols

Uniform data acquisition protocols are critical for reducing physiological and procedural variability. Cardiac gating should be considered mandatory, with synchronization achieved via pulse oximetry or electrocardiography (ECG). Standardized fixation targets and pupil dilation protocols should be implemented to ensure consistent imaging conditions. Acquisition duration should capture at least 3–5 cardiac cycles (approximately 4–6 seconds) to allow reliable hemodynamic assessment. Furthermore, a dark adaptation period of 5–10 minutes prior to imaging is recommended to stabilize choroidal vascular tone.

#### 4.5.3 Image processing and reporting

Consistency in image analysis and reporting will enhance transparency and reproducibility. Reconstruction algorithms, including phase unwrapping and motion correction, should ideally be made available as open-source tools. Automated segmentation methods should be validated against manual expert segmentation, with inter-grader reliability metrics reported. A standardized minimum dataset should include mean flow velocity, pulsatility index, resistivity index, and vessel diameter. All quantitative results should be reported using consistent units mm/s for velocity and μm for vessel diameter along with appropriate confidence intervals.

#### 4.5.4 Quality assurance

Robust quality assurance frameworks are necessary to ensure reliability across devices and study sites. The development and use of calibration phantoms with known flow velocities are recommended for inter-device validation. Studies should report test–retest repeatability, including coefficients of variation, to assess measurement stability. Finally, multicenter validation studies employing standardized protocols and centralized reading centers are strongly encouraged to support broader clinical adoption and regulatory acceptance.

Establishing open-access repositories for LDH data, following FAIR (Findable, Accessible, Interoperable, Reusable) principles, would facilitate meta-research and enable benchmarking between laboratories. Recommended data elements include raw holograms (where feasible), processed phase maps, extracted flow waveforms, and demographic/clinical metadata.
